# Involvement of Dihydrolipoyl Dehydrogenase in the Phagocytosis and Killing of *Paracoccidioides brasiliensis* by Macrophages

**DOI:** 10.3389/fmicb.2017.01803

**Published:** 2017-09-20

**Authors:** Taise N. Landgraf, Marcelo V. Costa, Aline F. Oliveira, Wander C. Ribeiro, Ademilson Panunto-Castelo, Fabrício F. Fernandes

**Affiliations:** ^1^Department of Biochemistry and Immunology, Ribeirão Preto Medical School, University of São Paulo Ribeirão Preto, Brazil; ^2^Department of Biology, Faculty of Philosophy, Sciences and Letters of Ribeirão Preto, University of São Paulo Ribeirão Preto, Brazil; ^3^Department of Cell and Molecular Biology, Ribeirão Preto Medical School, University of São Paulo Ribeirão Preto, Brazil

**Keywords:** *Paracoccidioides brasiliensis*, dihydrolipoyl dehydrogenase, exoantigen, subcellular localization, microbicidal activity

## Abstract

*Paracoccidioides brasiliensis* and *Paracoccidioides lutzii* are fungi causing paracoccidioidomycosis (PCM), an autochthonous systemic mycosis found in Latin America. These microorganisms contain a multitude of molecules that may be associated with the complex interaction of the fungus with the host. Here, we identify the enzyme dihydrolipoyl dehydrogenase (DLD) as an exoantigen from *P. brasiliensis* (Pb18_Dld) by mass spectrometry. Interestingly, the DLD gene expression in yeast form showed higher expression levels than those in mycelial form and transitional phases. Pb18_Dld gene was cloned, and the recombinant protein (rPb18_Dld) was expressed and purified for subsequent studies and production of antibodies. Immunogold labeling and transmission electron microscopy revealed that the Pb18_Dld is also localized in mitochondria and cytoplasm of *P. brasiliensis*. Moreover, when macrophages were stimulated with rPb18Dld, there was an increase in the phagocytic and microbicidal activity of these cells, as compared with non-stimulated cells. These findings suggest that Pb18_Dld can be involved in the pathogen-host interaction, opening possibilities for studies of this protein in PCM.

## Introduction

Paracoccidioidomycosis (PCM) is a chronic granulomatous mycosis caused by thermally dimorphic fungi *Paracoccidioides lutzii* and *P. brasiliensis*, which contain a complex of at least four different cryptic species, S1, PS2, PS3, and PS4 (Matute et al., 2006; Carrero et al., 2008; Teixeira et al., 2009, 2014; Theodoro et al., 2012). PCM is the most important systemic mycosis in Latin America with high prevalence in Brazil, Colombia, Argentina, and Venezuela. The mean annual mortality rate is 1.65 per million inhabitants, the highest for any systemic mycosis, making PCM the eighth most important cause of mortality among chronic or recurrent infectious, parasitic diseases, and infectious diseases in Brazil (Coutinho et al., 2002; Colombo et al., 2011; Bocca et al., 2013).

Fungi of the genus *Paracoccidioides* are asexual and dimorphic, growing as mycelial forms at 25°C and as yeast form at 37°C (da Silva et al., 1994; de Almeida, 2005). Transformation of the fungus from mycelia to yeast is responsible for establishing the infectious process (Bagagli et al., 2008) as well as for the initial interaction of the fungus with the immune system, which determines the fate of the infecting forms. Secretion of antimicrobial proteins by the pulmonary epithelium and phagocytic activity of resident alveolar macrophages mediates the initial response to infection (Calich et al., 2008). Infections can stimulate different subsets of T cells and consequently induce the production of distinct patterns of cytokines that are responsible for the protection or susceptibility of the host to pathogens (Della Bella et al., 2017), including fungal infections (Calich and Kashino, 1998; Oliveira et al., 2002; Romani, 2004).

The ability of this pathogenic fungus to develop a multi-faceted response to a wide variety of stressors found in the host is extremely important for its virulence and pathogenesis. Many of these extracellular molecules are secreted by or are associated with the fungal wall (Casotto, 1990; Diniz et al., 1999, 2001). Numerous proteins or glycoproteins that are common to different strains of *P. brasiliensis* are secreted in culture, and these are recognized, to varying degrees, by the serum of the patients (Panunto-Castelo et al., 2003). The most studied exoantigen (ExoAg) of *P. brasiliensis* is the 43-kDa glycoprotein (gp43), which is a molecule involved in binding of *P. brasiliensis* to the extracellular matrix and surface of epithelial cells (Vicentini et al., 1994; Gesztesi et al., 1996). More recently, Torres et al. (2013) showed that *P. brasiliensis* cell lines in which gp43 was silenced were less internalized by macrophages.

Some studies have been carried out in order to elucidate the complex programs of gene expression that *Paracoccidioides* spp. uses to survive when exposed to conditions similar to those found in the host. Tavares et al. (2007) showed, for instance, that *P. brasiliensis* regulates expression of 119 genes during phagocytosis. These genes were first associated with glucose and amino acid limitation, cell wall building, and oxidative stress. Subsequent studies also elucidated the molecules involved in the adaptation of the fungus. In addition to the activation of enzymes involved in the defense against oxidative stress (Derengowski et al., 2008; de Arruda Grossklaus et al., 2013; Parente-Rocha et al., 2015), other metabolic modifications have been suggested to occur in yeast cells, such as gluconeogenesis, ethanol production, and degradation of fatty acids and amino acids (Lima et al., 2014).

In this study, we aimed to identify the proteins that were secreted/excreted by *P. brasiliensis*. We also attempted to evaluate whether these molecules could have any association with the phagocytosis and killing activity, contributing to the pathogenic process. Although *P. brasiliensis* secretes a number of different proteins into the basal medium, one protein in particular drew our attention because it was present in high amount. The protein was identified as dihydrolipoyl dehydrogenase (Pb18_Dld), which was expressed at high levels in the pathogenic form, and was located in and outside the mitochondria, which improved the macrophage-mediated phagocytic and microbicidal activity.

## Materials and methods

### Ethics statement and mice

This work was conducted according to the ethical principles of animal research adopted by the Brazilian Society of Laboratory Animal Science, and received approval from the Ethics Committee on Animal Use of the Ribeirão Preto Medical School, USP. The Protocol number is 105/2007. Male BALB/c mice, aged 6–8 weeks, were purchased from the Central Animal House of the Ribeirão Preto Campus of University de São Paulo (USP), and were maintained in the Animal Facilities of the Department of Biology, Faculty of Philosophy, Sciences and Letters of Ribeirão Preto, USP.

### Growth conditions of the *P. brasiliensis* isolate

Virulent yeast cells of *P. brasiliensis* strain 18 (Pb18) were cultured for 15 days at 37°C in DMEM (Sigma-Aldrich, St Louis, USA), in a shaking incubator at 100 rpm. The yeast cells were maintained by culturing at 37°C, while the mycelial forms were obtained by culturing at 25°C. Both cultures were maintained under these conditions for 7 days. The transition phase from mycelium-to-yeast was induced by culturing the mycelia at 25°C for 72 h, changing the temperature to 37°C, and incubating for 24 h. The transition from yeast to mycelium was induced by growing yeast for 72 h at 37°C, changing the temperature to 25°C, and incubating for 24 h. One aliquot of each *P. brasiliensis* culture was treated with 50 μg/mL Calcofluor white stain (Sigma-Aldrich) for 30 min at 37°C to analyze the fungal morphological forms by fluorescence microscopy.

### Preparation of extracellular extract and total soluble antigen from *P. brasiliensis* and Pb18_Dld identification

Supernatants of the cultures containing ExoAgs were recovered by centrifugation at 10,000 × g, concentrated and diafiltrated against 10 mM phosphate-buffered saline (PBS, pH 7.2) using centrifugal filtration devices with a molecular weight cut-off of 10,000 kDa (Millipore, Billerica, USA). The ExoAgs were analyzed using 12% sodium dodecyl sulfate-polyacrylamide gel electrophoresis (SDS-PAGE). The gels were stained with Coomassie brilliant blue G250 (USB Corporation, Cleveland, USA). The relative amount of the 60-kDa protein band was calculated with the ImageJ 1.37v software (National Institutes of Health, Bethesda, USA) and the values were normalized against the total amount of loaded proteins. Next, the 60-kDa band was excised from the gel, and mass spectrometry was performed at the FingerPrint Proteomics and Mass Spectrometry Facility, College of Life Sciences, University of Dundee. Mascot (version 2.3; Matrix, United Kingdom) analysis was performed to identify peptides and to search for proteins in the NCBI non-redundant (nr) database. Total soluble antigen derived from *P. brasiliensis* yeast cells was prepared according to reported by Fernandes et al. (Panunto-Castelo et al., 2003).

### *In Silico* analysis

The *in silico* analyses were performed based on Pb18_Dld amino acid sequence (GenBank accession no. EEH50415.1) that was submitted to NetOGlyc 3.1 Server (http://www.cbs.dtu.dk/services/NetOGlyc-3.1/), NetNGlyc 1.0 Server (http://www.cbs.dtu.dk/services/NetNGlyc/), SignalP 4.1 Server (http://www.cbs.dtu.dk/services/SignalP/), and TargetP v1.1 Server (http://www.cbs.dtu.dk/services/TargetP/). Protein conserved domains are available in the CDD NCBI's conserved domain database (https://www.ncbi.nlm.nih.gov/Structure/cdd/wrpsb.cgi).

### Differential expression of dld mRNA from different morphological forms of *P. brasiliensis*

The gene expression profile of *P. brasiliensis* was analyzed in mycelium, yeast, and transition forms from mycelium-to-yeast and yeast-to-mycelium, which were cultivated as described previously. The total RNA from the *P. brasiliensis* morphological forms was extracted using *TRIzol* (Thermo Fisher Scientific Inc., Waltham, USA). Briefly, cells were harvested, disrupted by grinding in liquid nitrogen, and mixed with TRizol, according to the manufacturer's instructions. Total RNA was treated with DNase I (Thermo Fisher Scientific Inc.). The concentration of RNA was measured with the help of a spectrophotometer at 260 nm and the quality of RNA was evaluated by the ratio of absorbance at 260 and 280 nm. In order to ensure RNA integrity, samples were electrophoresed in 1.5% agarose gel. In addition, the absence of contaminating chromosomal DNA was confirmed, after the treatment of the RNA with DNaseI, by the absence of the HSP60 gene amplification (Fernandes et al., 2016) after PCR. cDNA was synthesized using 1 μg of total RNA with oligo-dT_12−18_ primer (Thermo Fisher Scientific Inc.) and SuperScript III reverse transcriptase (Thermo Fisher Scientific Inc.), according to the manufacturer's instructions. Real-time PCR was performed using *Kit Platinum SYBR Green qPCR SuperMix-UDG with ROX* (Thermo Fisher Scientific Inc.), according to the manufacturer's instructions. Next, specific primers were used for Pb18_DLD: 5′-GGTTTGGACAAGGTCGG-3′ (sense) and 5′-CATGGCCGTAGCCCTTC-3′ (antisense). To measure the gene expression levels, we used CFX96 real-time PCR detection system (Bio-Rad, Hercules, USA). Fold changes in mRNA expression were calculated using the 2^−ΔCq^ formula, where ΔCq is the difference in the threshold cycle (*Cq*) between the DLD (target) gene and the β-actin (Sequence ID: ref |XM_010763641.1|) or α-tubulin references (housekeeping) genes (Goldman et al., 2003). The reaction of the genes β-actin and α-tubulin was done using a couple of primers: 5′-GGATGAGGAGATGGATTATGG-3′ (sense) and 5′-GAAACACTCGACGCACACGAC-3′ (antisense); and 5′-GTGGACCAGGTGATCGATGT-3′ (sense) and 5′-ACCCTGGAGGCAGTCACA-3′ (antisense), respectively. All experiments were performed using biological and experimental triplicates.

### Cloning of the Pb18_Dld gene transcript

The cDNA obtained from the mRNA of yeast cells of *P. brasiliensis* was used to amplify the region encoding Pb18_Dld using oligonucleotide primers 5′-CATATGTTTCGGCCCCTTCTCCC-3′ (sense) and 5′-GGATCCCTAGAAATGAATCGCTTTCG-3′ (antisense), and high-fidelity Taq DNA polymerase (Thermo Fisher Scientific Inc.). The amplified fragment was cloned into pCR 2.1-TOPO vector (Thermo Fisher Scientific Inc.), and was sequenced at the Laboratory of Plant Molecular Biology from the *Faculty of Philosophy, Sciences, and Letters of Ribeirão* Preto—USP. Next, the fragment was removed from the pCR 2.1-TOPO vector using restriction enzymes *NdeI* and *BamHI* (Fermentas, Carlsbad, USA), and was sub-cloned into pET-28a vector (Addgene, Cambridge, USA).

### Expression and purification of rPb18_Dld

The pET-28a vector, containing the gene transcript of Pb18_Dld, was transformed into *Escherichia coli* BL21 cells for expression of the Pb18_Dld recombinant protein (rPb18_Dld). Briefly, cultures were grown in LB medium supplemented with kanamycin sulfate (50 μg/mL) at 37°C until an absorbance of 0.4–0.5 was observed at 600 nm. Then, rPb18_rDld expression was induced with 0.8 mM isopropyl-D-thiogalactopyranoside (IPTG) for 6 h. The bacterial cells were then harvested by centrifugation at 10,000 × g, at 25°C for 10 min, resuspended in buffer containing 50 mM NaH_2_PO_4_, 300 mM NaCl, and 20 mM imidazole (pH 8.0), and lysed by sonication. The pellet containing the insoluble fractions was washed five times with a washing buffer [50 mM NaH_2_PO_4_, 300 mM NaCl, 2 M urea, 2 mM 2-mercaptoethanol, 20 mM imidazole, and 1% Triton X-100 (pH 8.0)]. Then, the pellet was resuspended in solubilization buffer [50 mM NaH_2_PO_4_, 300 mM NaCl, 20 mM imidazole, 7 M urea (pH 8.0)], followed by incubation for 1 h at room temperature. The solubilized material was recovered by centrifugation at 10,000 × g at 4°C for 10 min, and was filtered through Millex-GV PVDF (pore size, 0.22 μm; Millipore). The rPb18_Dld was purified by performing metal chelate affinity chromatography with a Ni^2+^–Sepharose affinity column (His-Trap; GE Healthcare, Piscataway, USA). Next, the rPb18_Dld was eluted using elution buffer (50 mM NaH_2_PO_4_, 300 mM NaCl, 300 mM imidazole, 7 M urea), refolded by dialysis against PBS, and concentrated by ultrafiltration. Protein concentration was determined using BCA kit (Pierce Chemical Co., Rockford, USA) and purity, size, and identity of the rPb18_Dld protein were evaluated using SDS-PAGE (12%). In addition, the recombinant protein samples were analyzed for endotoxin contamination by using Limulus amebocyte lysate (LAL) assay (Sigma-Aldrich), and were found to contain < 0.05 ng/mL of bacterial endotoxin.

### Mice immunization

We used purified rPb18_Dld protein to generate specific anti-rPb18_Dld polyclonal antibody in mice serum. Male BALB/c mice were immunized by subcutaneous injection of 50 μL of purified protein (10 μg/mice) with 50 μL of complete Freund's adjuvant (CFA, Sigma-Aldrich) at first administration. Animals were boosted twice, at 1-week intervals, with the same amount of antigen emulsified in 50 μL of incomplete Freund's adjuvant. One week after the last boost, the serum containing polyclonal antibody to rPb18_rDld was aliquoted and stored at −20°C.

### Western blotting

The electrophoresis analyses were performed on 12% SDS–PAGE, and transferred to polyvinylidene fluoride membranes (PVDF)—Hybond membranes Amersham 0.45 P PVDF (GE Healthcare). Thereafter, membranes were incubated with Tris-buffered saline (TBS-T) [20 mM Tris-HCl, 150 mM NaCl and 0.1% (v/v) Tween-20 (pH 7.6)] containing 3% gelatin for 1 h at 25°C, and incubated for 4 h at room temperature with anti-Pb18_rDld polyclonal antibody diluted 1:1,000 in TBS-T containing 1% gelatin. Thereafter, membranes were washed five times with TBS-T and incubated for 2 h at room temperature with the anti-mouse IgG rabbit secondary antibody conjugated to peroxidase (Sigma-Aldrich) diluted 1:500 in TBS-T containing 1% gelatin. Blots were washed five times with TBS-T and then immersed in a fresh mixture of 4-chloro-1-naphthol (4C1N) (Sigma-Aldrich) and 3′3-diaminobenzidine tetrahydrochloride (Sigma-Aldrich) or ECL reagent for 1 min to detection of protein using a ChemiDoc MP Imaging System (Bio-Rad, Hercules, USA).

### Electron microscopy

The Pb18_Dld subcellular localization was performed with yeast and mycelia washed with PBS and fixed in 3.7% formaldehyde buffered with PBS at 25°C for 1 h. After fixation, cells were washed with PBS and resuspended in 0.05% glutaraldehyde buffered with PBS and incubated at 25°C. After 15 min, the cells were centrifuged at 5,000 × g for 5 min at 25°C and washed three times with 1 mL of PBS. Then, the cells were resuspended in PBS containing 0.1% Triton X-100 and incubated at 25°C for 45 min. The cells were recovered by centrifugation at 5,000 × *g* for 5 min and washed with 1 mL PBS. Samples were incubated with 1 mL PBS-1% BSA at 25°C. After 45 min, anti-Pb18_rDld polyclonal antibody (1:500) was added for 1 h, followed by incubation at 25°C under gentle agitation. After washing with PBS-1% BSA, colloidal gold-conjugated anti-mouse IgG (Nanoprobes Inc., NY, USA) was added to the cells for 1 h at 25°C under gentle agitation. The cells were washed five times with PBS, with 5 min incubation for each wash and fixed with 2.5% glutaraldehyde in 100 mM cacodylate buffer (pH 7.4) (EM Sciences, Hatfield, USA) at 25°C for 1 h. Cells were washed with cacodylate buffer and incubated at 4°C for 18 h, followed by washing five times with ultrapure water. Further processing of cells was conducted as per the manufacturer's instructions of GoldEnhancement (Nanoprobes). Subsequently, the fixed cells were incubated in 1% OsO_4_ (EM Sciences) for 2 h, rinsed in ultrapure water, and incubated with a solution of saturated thiocarbohydrazide (EM Sciences), followed by 1% OsO_4_. Yeast cells were dehydrated with ethanol (30–100% v/v) and coated with gold in a BAL-TEC SCD 050 Sputter Coater (BAL-TEC AG, Balzers, Liechtenstein). Finally, the sections were observed with a transmission electron microscope Jeol JEM—100 CXII (JEOL, Peabody, MA, USA).

### Macrophages phagocytic and microbicidal activity assays

Macrophages were obtained from the peritoneal cavity of BALB/c mice 72 h after an intraperitoneal injection of 1 mL of 3% sterile sodium thioglycollate medium (Sigma-Aldrich), according to the described protocol (Freitas et al., 2016). Peritoneal macrophages (1 × 10^6^) were stimulated or not with 10 μg of rPb18_Dld and maintained in a 5% CO_2_ humidified atmosphere incubator at 37°C. After 24 h, macrophages were infected with 3 × 10^5^ yeast cells of *P. brasiliensis* and incubated for 4 h at 37°C. The cultures were washed with RPMI-incomplete medium. To evaluate the phagocytic activity, 1 mL of distilled water was added to the microplate wells to promote macrophage lysis. The samples were serially diluted and cultured for 10 days at 37°C in solid BHI medium supplemented with 1% glucose to determine the presence of colony forming units (CFU) of viable yeasts. To analyze microbicidal activity, the washed cultures were additionally incubated for 48 h. Macrophage lysis and yeast cultures were carried out as described above. The number of CFU were counted and expressed as CFU/well.

### Statistical analyses

Data are expressed as mean ± standard deviation from at least duplicate experiments. Statistical differences between the means of the experimental groups were determined using the Student's unpaired *t*-test (GraphPad Software, San Diego, USA). Differences with *P* < 0.05 were considered statistically significant.

## Results

### Identification of dihydrolipoyl dehydrogenase (Pb18_Dld) as the predominant protein in the *P. brasiliensis* culture supernatant

**Figure 1:**
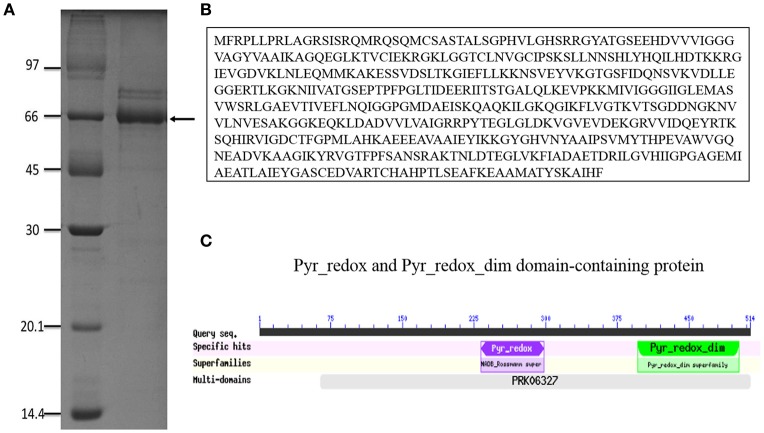
Pb18_Dld identification. **(A)** SDS-PAGE result of *P. brasiliensis* exoantigens grown in DMEM, for 15 days at 37°C. After this period, cultures were centrifuged, and the supernatant was dialyzed and analyzed. The band indicated by the arrow was excised from the gel and analyzed by mass spectrometry. SDS-PAGE was stained with Coomassie brilliant blue. **(B)** Pb18_Dld amino acid sequence. **(C)** Conserved domains (available on CDD NCBI's conserved domain database).

Antigens secreted by *P. brasiliensis* could play an important role in fungus-host interactions. Therefore, we sought to identify new antigens from supernatants after centrifugation of *P. brasiliensis* cultured in DMEM, which is a basal medium that does not contain proteins. When the supernatants were analyzed by SDS-PAGE, we observed that a major band of 60 kDa was invariably present in all ExoAgs preparations (Figure 1A), comprising more than 85% of the total protein content. Some minor bands were observed in the supernatants, such as the proteins of 90-, 85-, and 43-kDa, the latter possibly being gp43. One-dimensional nano-LC-MS/MS analysis of tryptic fragments of the 60 kDa protein band revealed two unique peptides that matched the sequence of Pb18_Dld (GenBank accession number EEH50415.1). Pb18_Dld is a protein involved in fungal metabolism (Weber et al., 2012; Parente-Rocha et al., 2015), and is composed of 514 amino acids (Figure 1B), with a small NADH binding domain, a larger FAD-binding domain (Pyr_redox), and a dimerization domain (Pyr_redox_dim) (Figure 1C).

**Figure 2:**
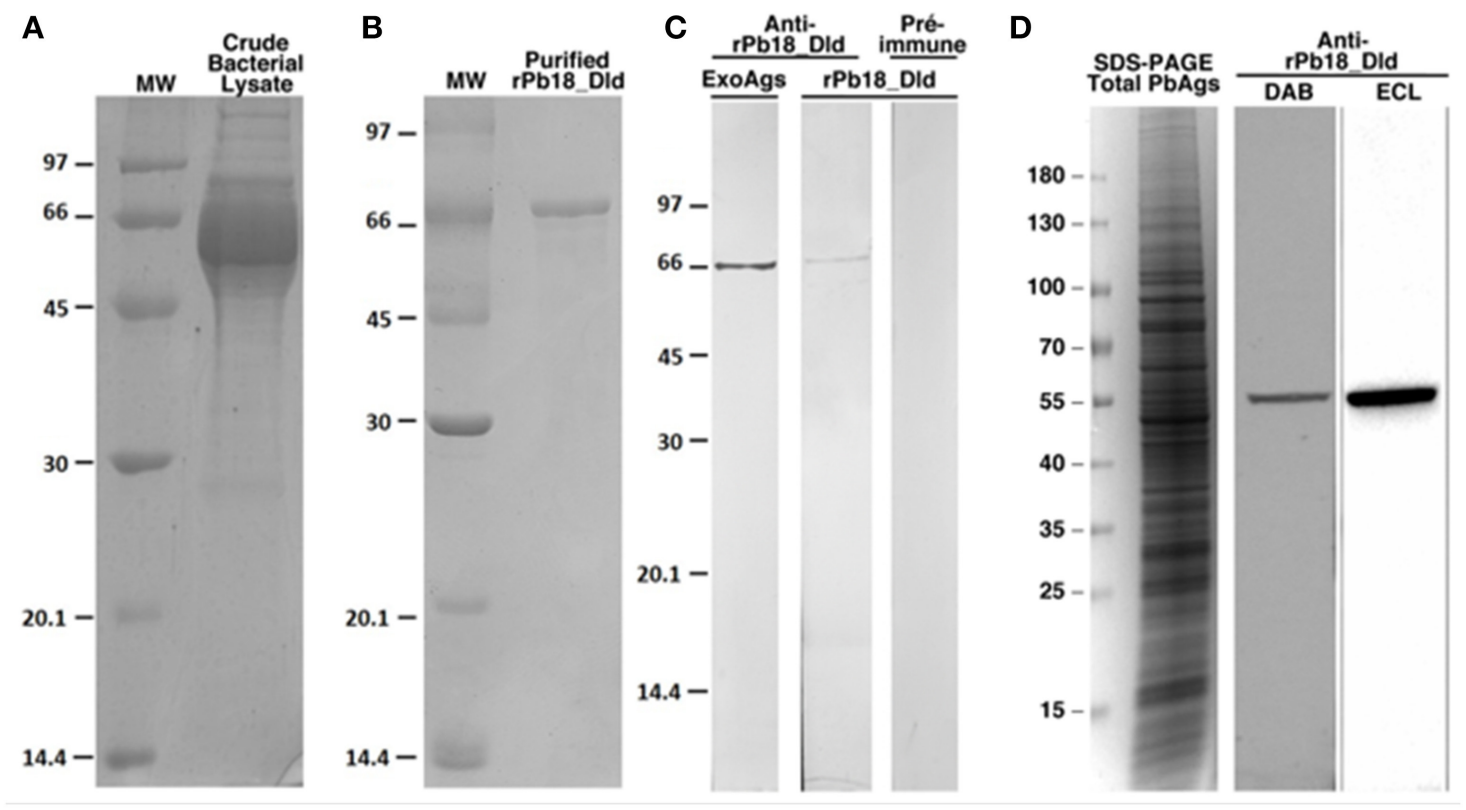
Analysis of the expression and purification of rPb18_Dld, and production of anti-rPB18_Dld polyclonal antibody. SDS-PAGE results of **(A)** rPb18_Dld expression and **(B)** purified rPb18_Dld by affinity chromatography using a liquid chromatographic system, Akta Purifier in HisTrap Crude FF column. The purified protein was dialyzed against PBS. SDS-PAGE was stained with Coomassie brilliant blue. **(C)** Western blotting analysis of anti-rPb18_Dld polyclonal serum. Membranes containing rPb18_Dld or total exoantigens containing native Pb18_Dld (ExoAg) were incubated with serum containing polyclonal anti-rPb18_Dld antibodies. Negative control of the reaction was performed using the membrane containing rPb18_Dld incubated with preimmune serum. The membranes were visualized by peroxide substrate hydrogen and diaminobenzidine tetrahydrochloride. **(D)** SDS-PAGE stained with Coomassie brilliant blue of total soluble antigen from *P. brasiliensis* (Total PbAgs). Western blotting analysis of anti-rPb18_Dld polyclonal serum with membranes containing Total PbAgs were incubated with 1:1,000 containing polyclonal anti-rPb18_Dld antibodies. The membranes were visualized by peroxide substrate hydrogen and diaminobenzidine tetrahydrochloride (DAB) or by chemiluminescent with ECL reagent (ECL).

To confirm the presence of Pb18_DLD in the culture supernatant, we used anti-rPb18_Dld polyclonal antibody. First, we cloned the *Pb18_Dld* gene transcript in an expression plasmid, pET28-a, for the production of the recombinant protein in bacteria (rPb18_Dld) (Figure 2A). Figure 2B shows the recombinant protein, which was purified using a nickel column for the production of anti-rPb18_Dld polyclonal antibodies in mouse. Use of the anti-recombinant protein antibody revealed the presence of Dld in total soluble antigen from yeast cells, as well as, in *P. brasiliensis* DMEM culture supernatant (Figures 2C,D), suggesting that Pb18_Dld is a protein secreted by *P. brasiliensis*.

### *In Silico* analysis of Pb18_Dld

**Figure 3:**
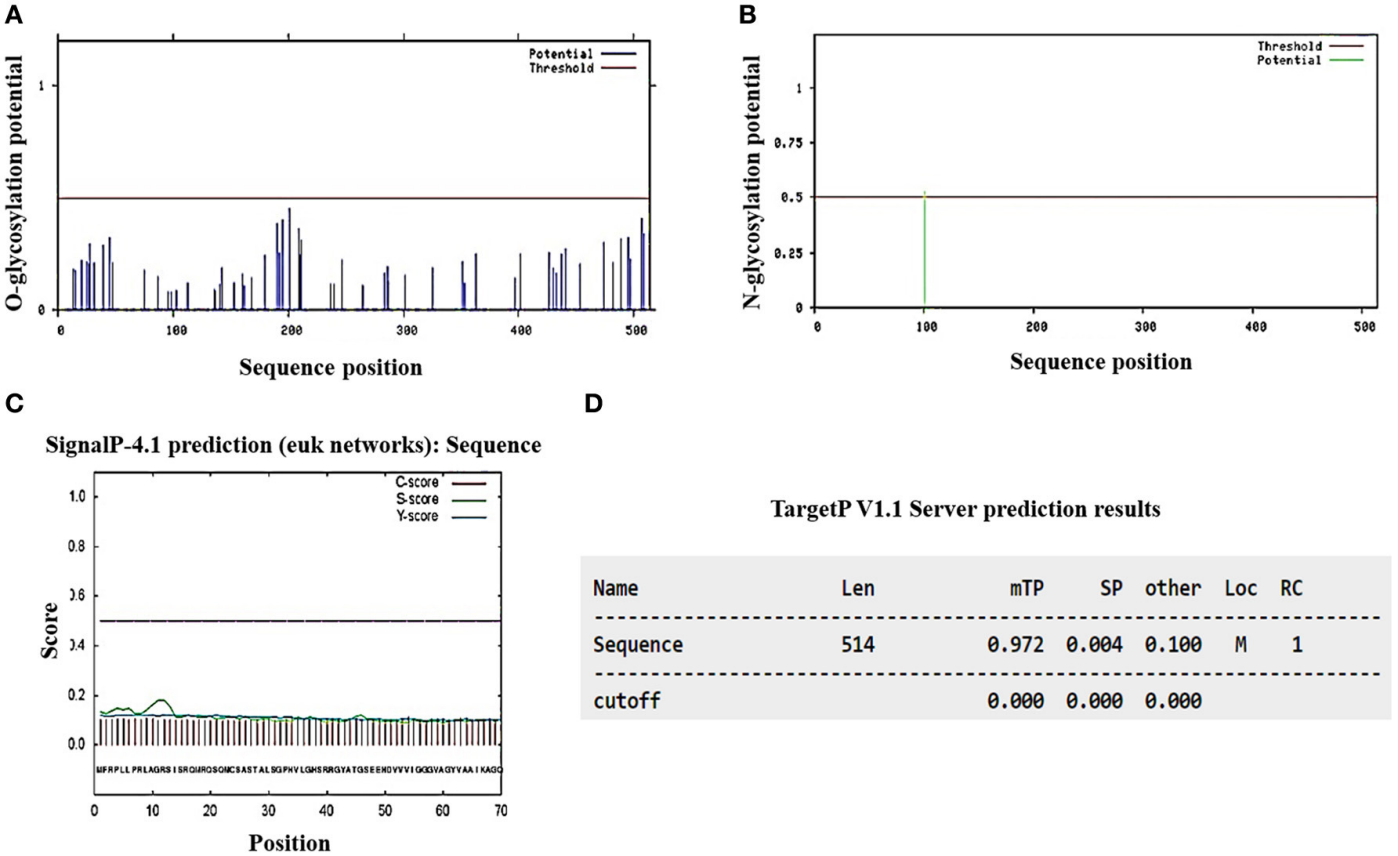
*In silico* analysis of the presence of glycosylation potential sites, signal peptide, and prediction analysis of subcellular location from Pb18_Dld. The amino acid sequence of Pb18_Dld protein was used for analysis in different programs **(A,B)** NetOGlyc 1.0 and NetNGlyc 3.11 show graphics with horizontal lines that represent the reliability limits of the results and vertical lines that indicate the potential sites of O- and N-glycosylation. **(C)** SignalP-4.1 shows graphic with dotted line, representing the reliability limits of results. The lines C, S, and Y-score indicate possible signal peptide cleavage positions and signal peptide regions. **(D)** TargetP v1.1 shows the length (Len), mitochondrial target peptide (mTP), secretory pathway (SP), localization (Loc), mitochondria (M), and reliability class (RC).

As part of a complementary analysis to characterize Pb18_Dld, we conducted an *in silico* examination of the protein to check for the presence of putative N- and O-linked oligosaccharides and signal peptides in the amino acid sequence. The analysis revealed that this protein possessed only one putative N-glycosylation site in the asparagine residue at the 101 position, no potential site of O-glycosylation (Figures 3A,B) and no signal peptide that targeted the protein to the secretory pathway (Figures 3C,D). Moreover, the data obtained *in silico* by using TargetP predicted that Pb18_Dld was localized in mitochondria (Figure 3D), although it was also identified in the extracellular fraction. Together, these data suggest that Pb18_Dld is a mitochondrial protein that can be secreted via a non-conventional pathway.

### Pb18_Dld from *P. brasiliensis* is expressed primarily in yeast form

**Figure 4:**
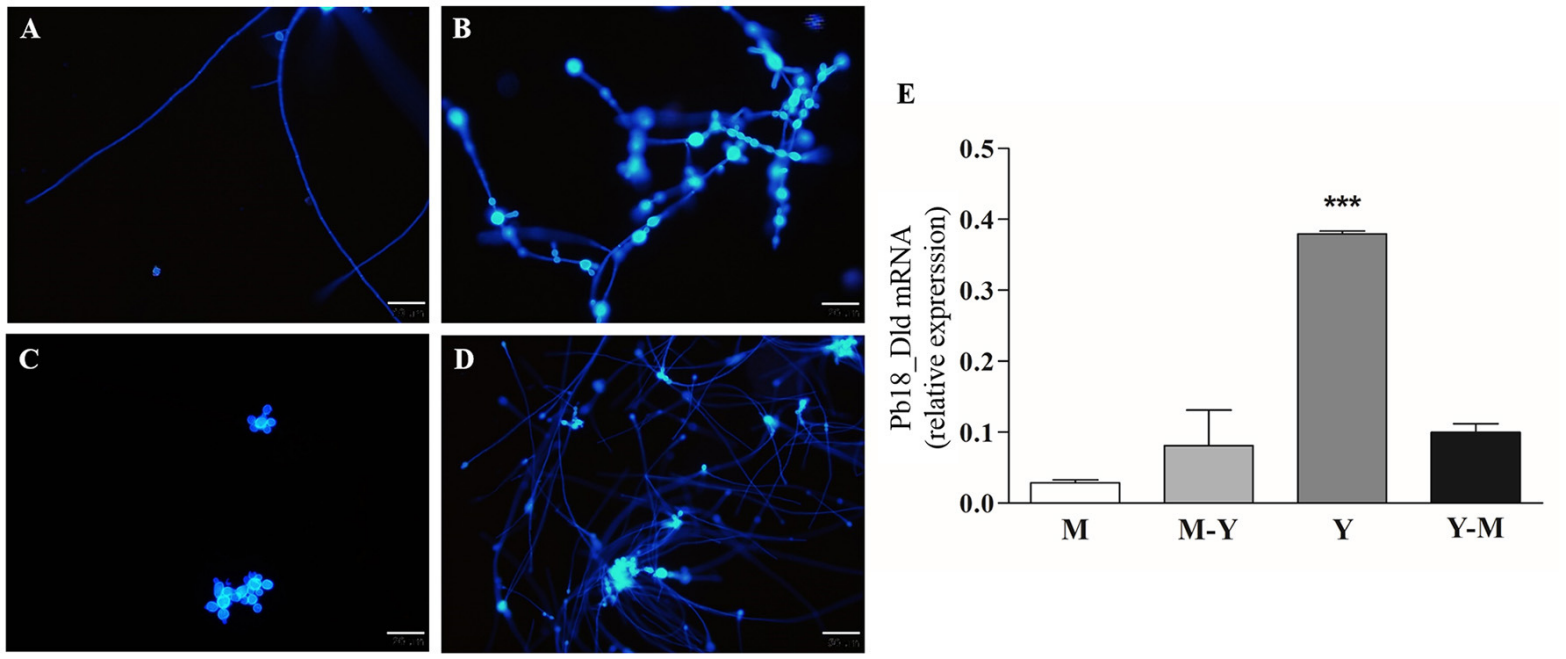
Pb18_Dld mRNA differential expression in different morphological forms of *P. brasiliensis*. **(A–D)** Morphological analysis by fluorescence microscopy. An aliquot of each fungal culture was incubated with Calcofluor white stain solution (50 μg/mL) for labeling the fungal cells. The bars correspond to 20 μm. **(E)** Evaluation of Pb18_Dld mRNA differential expression by RT-qPCR using cDNA from mycelia **(A)**, mycelia-to-yeast **(B)**, yeast **(C)**, and yeast-to-mycelia **(D)**. The β-actin and α-tubulin reference genes were used as endogenous controls to normalize the relative Pb18_Dld mRNA expression.

To determine if there were differences in expression levels of Pb18_Dld in yeast, mycelia, and transition forms, we compared normalized mRNA-expression levels among *in vitro* grown phases. Prior to mRNA extraction, we confirmed that the fungal morphology corresponded to expected forms by fluorescence microscopy of samples stained with Calcofluor white (Figures 4A–D). Pb18_Dld transcripts were detected in all evaluated morphological forms, although they showed higher expression in yeast, which is the pathogenic form, as compared with levels in transition phases (mycelium-to-yeast and yeast-to-mycelium) or the mycelial form (Figure 4E).

### Pb18_Dld subcellular localization in *P. brasiliensis* yeast and mycelia

**Figure 5:**
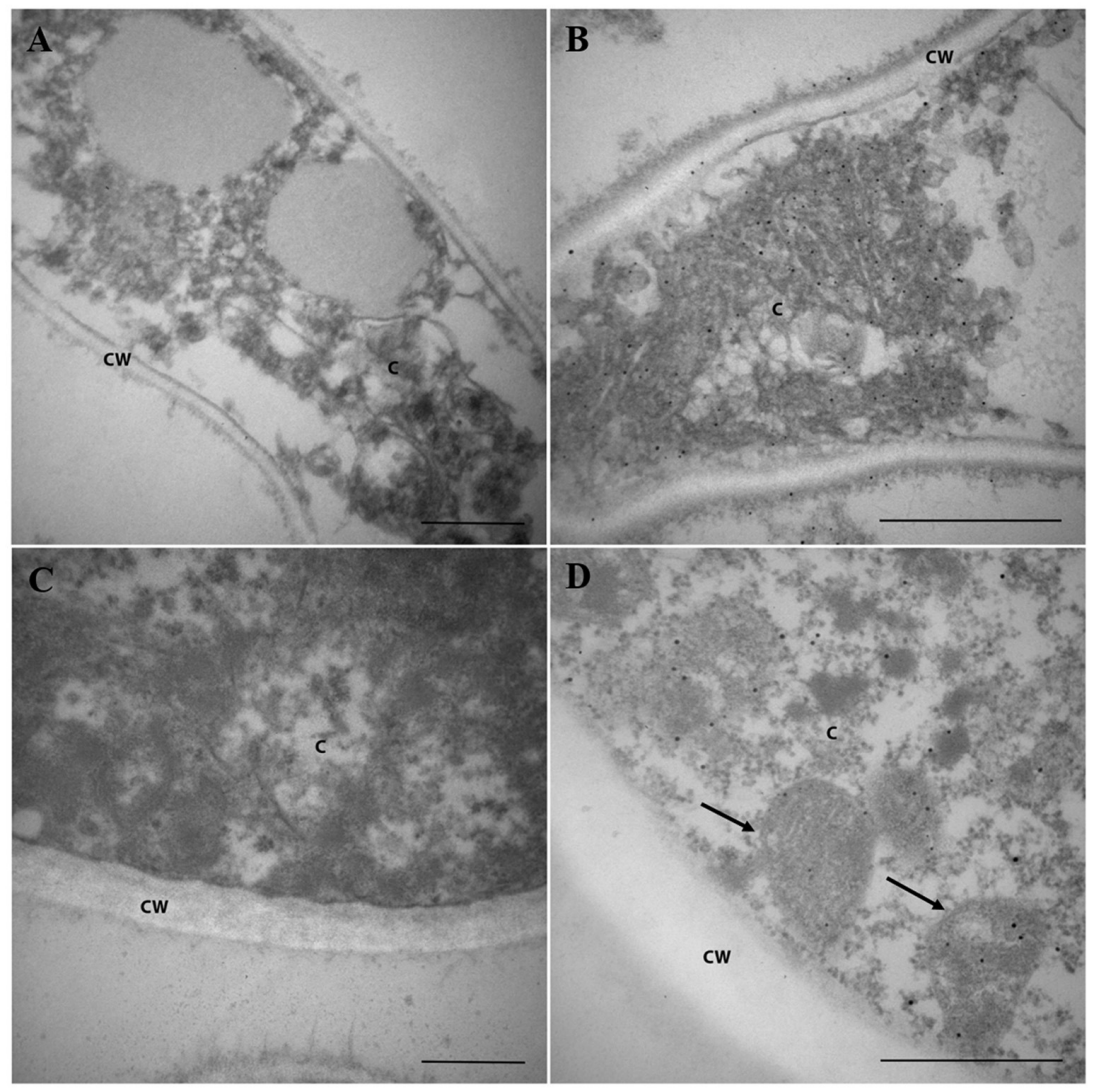
Pb18_Dld localization in mycelia and yeast cells from *P. brasiliensis*, visualized by immunoelectron microscopy. **(A,B)** Mycelium and **(C,D)** yeast from *P. brasiliensis* were labeled with primary polyclonal antibody anti-rPb18_Dld **(B,D)** or pre-immune serum **(A,B)** and secondary antibody conjugated to colloidal gold. The staining of Pb18_Dld was analyzed by electron microscopy. Bars correspond to 2 μm. The C and CW identifications correspond to the cytoplasm and cell wall, respectively. The arrows indicate mitochondria.

As Pb18_Dld was a component of ExoAgs and *in silico* analysis of its sequence did not show the signal peptide, we intended to investigate the localization of Pb18_Dld in fungal yeast. Yeast and mycelia of *P. brasiliensis* were fixed and incubated with anti-rPb18_Dld polyclonal mice antibody, and subsequently, incubated with secondary antibody, colloidal gold anti-mouse IgG conjugate, for immunodetection of the protein by electron microscopy. Control samples were obtained by incubation with pre-immune mice serum (Figures 5A,C). Electron microscopy assays revealed the ubiquitous distribution of gold particles in cytoplasm and mitochondria of both forms of *P. brasiliensis* (Figures 5B,D). In addition, the protein was also present in the mycelial cell wall (Figure 5B). Together, these results are in accordance with the mitochondrial localization predicted by *in silico* analysis, and suggest that Pb18_Dld could be secreted by an alternative secretion pathway.

### Phagocytosis of macrophages is increased in the presence of rPb18_Dld

**Figure 6:**
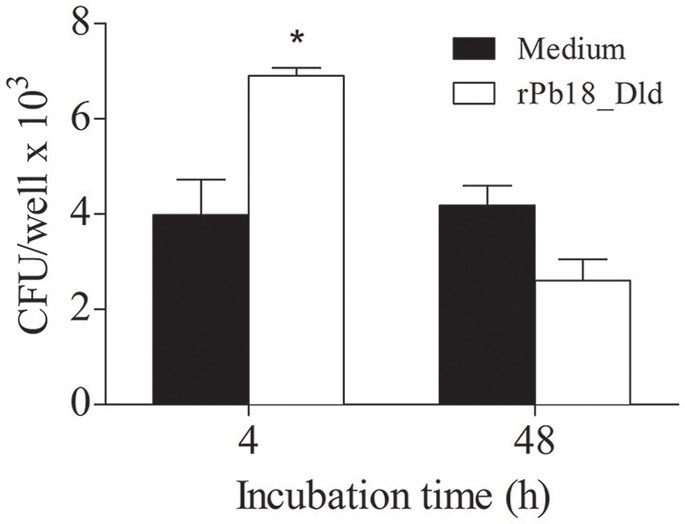
Phagocytic and microbicidal activity of macrophages stimulated with rPb18_Dld. *P. brasiliensis* yeast cells were grown in DMEM medium at 37°C for 5 days and co-cultured with peritoneal macrophages, prestimulated or not with 10 μg of rPb18_rDld. Phagocytic and microbicidal activity were determined by counting the CFU of yeast recovered from the lysate of murine peritoneal macrophages after 4 and 48 h of incubation with yeast at 37°C, respectively. Results represent the mean ± standard deviation values of two independent experiments in triplicate. ^*^P < 0.05 in relation to the other experimental groups, as determined by Student *t*-test.

Macrophages comprise one of the primary defense mechanisms against infection by *P. brasiliensis* (Brummer et al., 1988a,b). Additionally, Pb18_Dld showed higher expression in the pathogenic form. Therefore, we evaluated whether this protein could have some effect on the phagocytic and microbicidal activity of macrophages. Phagocytosis and killing assays were performed using peritoneal macrophages. Interestingly, when the cells were pretreated with rPb18_Dld, we observed a significant increase in the phagocytic activity, i.e., after 4 h of co-culture, from 3.98 × 10^3^ CFU/mL ± 1.28 to 6.9 × 10^3^ CFU/mL ± 0.30. We also observed an increase in the microbicidal activity i.e., after 48 h of co-culture, with a reduction from 4.18 × 10^3^ CFU/mL ± 0.71 to 2.6 × 10^3^ CFU/mL ± 0.78 at rDld-Pb18 stimulated-macrophages, representing relative killing of 62% of yeast cells (Figure 6). Together, our data suggest that this protein may be important to improve fungicidal process by macrophages during infection.

## Discussion

In this study, to identify new antigens from *P. brasiliensis* that could be associated with the host, we focused on ExoAgs preparations and isolated a highly expressed protein, identified by mass spectrometry as Pb18_Dld. The fact that Pb18_Dld could be detected in the extracellular extract suggested that it was an ExoAg released by the fungus. This is an intriguing observation because orthologs of this protein were typically detected in the mitochondria. It is part of the machinery responsible for aerobic respiration, in which the pyruvate produced by glycolysis and via the phosphate pentose pathway is mainly oxidized and decarboxylated to acetyl-CoA by the pyruvate dehydrogenase complex (Lehninger et al., 2005). In accordance with its function, our electron microscopy data showed that this enzyme is located in the mitochondria and, interestingly, in the cytoplasm of mycelia and yeast and in the mycelial cell wall. Corroborating our findings, work published by Weber et al. (2012) showed that Dld from *P. lutzii* is a component of extracellular proteomics of mycelial and yeast forms.

The presence of Pb18_Dld and other proteins at the cell surface or outside the fungal cell wall, in the absence of a conventional N-terminal signal sequence responsible for targeting the protein to classical secretory pathway, is an intriguing question. To explain this occurrence, non-conventional secretory pathways, as well as unusual signal sequences, had been described. An example of unusual transport is the extracellular vesicles produced by fungi (Straus et al., 1996). In fact, some enzymes involved in the glycolytic pathway and tricarboxylic acid cycle were identified in the contents of these vesicles (Vallejo et al., 2012).

Carbohydrates are the primary and preferred carbon source used by most organisms (Askew et al., 2009). For survival of pathogens in the hostile host microenvironment, expression of virulence factors and their ability to assimilate various carbon sources for energy production are important (Lorenz et al., 2004; Barelle et al., 2006; Askew et al., 2009; Romani, 2011). Some studies indicate that metabolic remodeling observed in *Paracoccidioides* spp. infection is a survival strategy (Lima et al., 2014). *P. lutzii*, when grown under copper deprivation, showed induction of the pyruvate dehydrogenase complex, suggesting that availability of acetyl-CoA by the generated pyruvate could be important for the survival of *P. lutzii* in inhospitable conditions (de Oliveira et al., 2014). Parente-Rocha et al. (2015) demonstrated that the fungi could modify their metabolism when subjected to conditions that mimic macrophage infection by *P. brasiliensis*. In this study, we observed that there is an increase in the Dld expression in yeast cells, which is the pathogenic form of *P. brasiliensis*.

High Dld expression in the ExoAgs preparation of *P. brasiliensis* raised the possibility that this protein might be a potential virulence factor, facilitating the infection. A common approach for recognition of virulence factors in dimorphic fungi is the correlation with increased expression of the possible factor in the pathogenic form of the fungus (Rappleye and Goldman, 2006). Our results have shown that the expression of Pb18_Dld mRNA is the highest in the yeast morphology, which is the fungal pathogenic form. Similar to Dld, glyceraldehyde-3-phosphate dehydrogenase (GAPDH), a glycolytic enzyme from *P. brasiliensis*, was shown to be present in larger amounts in the parasitic yeast phase than in the mycelia, suggesting a putative role for GAPDH in the parasitic phase of the fungus (Barbosa et al., 2004). Subsequently, it was demonstrated that GAPDH is located at the outermost layer in the cell wall of *P. brasiliensis* yeast forms, and has adhesive properties (Barbosa et al., 2006). The ability to perform biological functions unrelated to the protein canonical function is common for some metabolic enzymes of *Paracoccidioides* spp. that are frequently involved in virulence (Marcos et al., 2014). When we added rPb18_Dld to cultures of macrophages from the peritoneal cavity, it induced a higher phagocytosis rate. To date, there has been no report regarding the role of this metabolic protein in immune responses of the host. Although our results suggested that Pb18_Dld possibly displayed immunostimulatory effects in host cells, it was undeniable that many molecules induced the activation of macrophages and yet had a deleterious effect on the host (Medzhitov, 2008). Therefore, further experiments are needed before we reject the association of Pb18_Dld with *P. brasiliensis* virulence.

In summary, we demonstrated that Dld, a glycolytic enzyme, which is present in the mitochondria, cytoplasm, and cell wall and is secreted by *P. brasiliensis*, is more prominently expressed in the pathogenic form of the fungus. In addition, this protein stimulates the immune system by increasing the phagocytic and microbicidal activity of macrophages. Although the significance of this protein in disease progression has to be confirmed by virulence tests, for instance, using genetically Pb18_Dld deficient yeast, our work offers a new approach to studying Pb18_Dld as a potential therapeutic target in PCM.

## Author contributions

Conceived and designed the experiments: TL, AP, and FF. Performed the experiments: TL, MC, AO, WR, and FF. Analyzed the data: TL, AP, and FF. Contributed reagents/materials/analysis tools: AP and FF. Wrote the paper: TL, AP, and FF.

### Conflict of interest statement

The authors declare that the research was conducted in the absence of any commercial or financial relationships that could be construed as a potential conflict of interest. The reviewer SB and handling Editor declared their shared affiliation, and the handling Editor states that the process nevertheless met the standards of a fair and objective review.
